# Supplementary material for: Generation of Spike-Extracellular Vesicles (S-EVs) as a Tool to Mimic SARS-CoV-2 Interaction with Host Cells
Source: Cells. 2022 Jan 3;11(1):146. doi: 10.3390/cells11010146 (PMC8750506; doi:10.3390/cells11010146)
Supplement: Supplementary file 1 [file cells-11-00146-s001.zip › cells-1461934-supplementary.pdf]

Generation of spike- extracellular vesicles (S-EVs) as a tool to mimic SARS-CoV-2 interaction with host cells

Roberta Verta [1], Cristina Grange [2], Renata Skovronova [1], Adele Tanzi[1], Licia Peruzzi [3], Maria Chiara Deregibus[4], Giovanni Camussi[5] and Benedetta Bussolati [1,*]

1 Department of Molecular Biotechnology and Health Sciences, University of Turin, Via Nizza 52, 10126 Turin, Italy; roberta.verta@unito.it (R.V.); renata.skovronova@unito.it (R.S.); benedetta.bussolati@unito.it (B.B.)

2 Department of Medical Science; University of Turin, Via Nizza 52, 10126 Turin, Ital y; cristina.grange@unito.it (C.G.)

3 Pediatric Nephrology Unit, Regina, Margherita Children's Hospital, Città della Salute e della Scienza di Torino, Turin, Ita ly; licia.peruzzi@unito.it (L.P.)

4 2i3T Business Incubator and Technology Transfer; University of Turin, 10126 Turin, Italy; mariachiara.deregibus@unito.it (M.C.D.)

5 Department of Medical Sciences, University of Turin, Corso Dogliotti 14, 10126 Turin, Italy; giovan -ni.camussi@unito.it (G.C.)

Supplementary figures

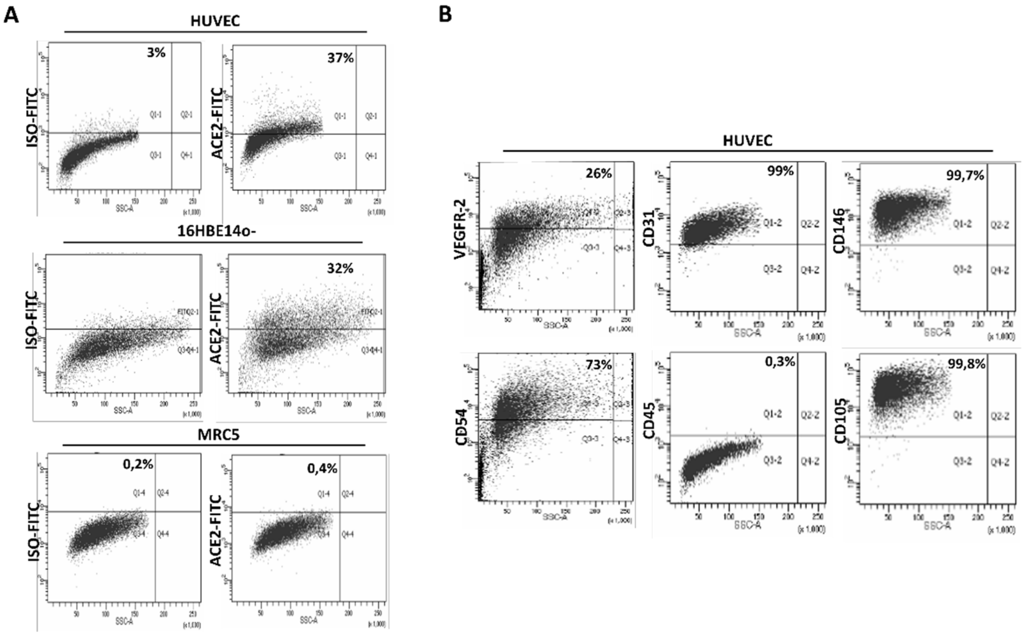

Supplementary Figure S1. Analysis of ACE2 expression and HUVEC characterization. (**A**) Representative flow cytometry analysis of human umbilical vein endothelial cells (HUVEC) , normal human bronchial epithelial cells (16HBE14o-) and human lung fibroblast cells (MRC5) showing the negative staining of a control isotype (ISO -FITC) in both type cells and the positive expression of ACE2 (ACE2-FITC) on HUVEC (37%) and 16HBE14o- cells (24%) respect to the negative control MRC5 (0,4%). (**B**) Characterization of HUVEC for the positive endothelial markers VEGFR, CD31, CD146, CD54 , CD105 and CD45 negative.

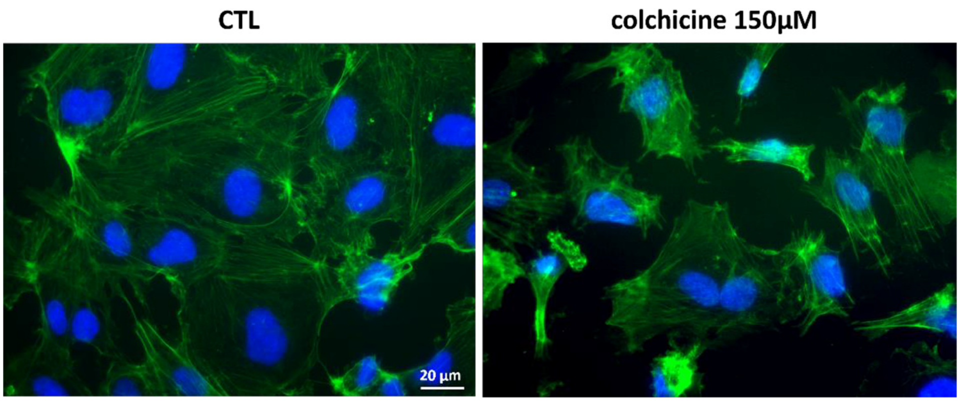

Supplementary Figure S2. Colchicine effect on HUVEC. Representative immunofluorescence images of HUVEC stained with phalloidin (green), nuclear stain DAPI (blue) in normal condition (CTL) and after 2 hours of colchicine treatment (colchicine 150μM), magnification 40×.
